# Laser in the Treatment of Atonic Wounds

**DOI:** 10.3390/biomedicines11071815

**Published:** 2023-06-24

**Authors:** Maur Sebastian Horgos, Ovidiu Laurean Pop, Mircea Sandor, Ioan Lucian Borza, Rodica Negrean, Felicia Marc, Klaudia Major, Liliana Sachelarie, Carmen Grierosu, Anca Huniadi

**Affiliations:** 1 Department of Surgical Disciplines, Faculty of Medicine and Pharmacy, University of Oradea, 1st December Square 10, 410073 Oradea, Romania; mauhorgos@yahoo.com (M.S.H.); ancahuniadi@gmail.com (A.H.); 2 Department of Pathology, County Clinical Emergency Hospital, Faculty of Medicine and Pharmacy, University of Oradea, 1 December Sq. No. 10, 410087 Oradea, Romania; drovipop@yahoo.com; 3 Department of Morphological Disciplines, Faculty of Medicine and Pharmacy, University of Oradea, 1st December Square 10, 410073 Oradea, Romania; borzaioanlucian@yahoo.com; 4 Department of Preclinical Disciplines, Faculty of Medicine and Pharmacy, University of Oradea, 1st December Square 10, 410073 Oradea, Romania; rodicanegrean@yahoo.com; 5 Department of Medical Disciplines, Faculty of Medicine and Pharmacy, University of Oradea, 1st December Square 10, 410073 Oradea, Romania; feliciamarc.dr@gmail.com; 6 Szabolcs-Szatmár-Bereg County Hospital and University Centre, Josa Andras, Szent István u. 68, 4400 Nyiregyhaza, Hungary; claudia_major1990@yahoo.com; 7 Department of Preclinical Disciplines, Faculty of Dental Medicine, Apollonia University, 700511 Iasi, Romania; grierosucarmen@yahoo.com

**Keywords:** wound, evolution, laser, therapy

## Abstract

Atonic wounds represent a major health problem, being frequently encountered in medical practice with consequences that have a negative impact on the patient’s daily life as well as their general condition. In this study, a brand laser with a 12-watt probe was used to stimulate patients’ wounds. We involved in this study a group of 65 patients, which was compared with a group of 30 patients, the latter not receiving this laser therapy. The data were accumulated from the questionnaire of subjective assessment of the laser impact on patients’ condition as well as from the local evolution. We noticed the improvement of the local symptomatology which was found to be more effective in the patients from the study group compared to the reference group. The beneficial and positive effects, mainly on the symptoms but also on the local evolution of atonic wounds, can be observed in our study. We consider that this therapy is of major importance considering the lower costs both from the shortening of hospitalization and the long-term use of various substances. The early reintegration of patients into daily life is an important benefit for them.

## 1. Introduction

A wound is considered chronic when it does not heal, either because of a slow healing process, or because of its recurrence after a certain time period [1]. Chronic wounds are those that have not fully recovered their normal anatomical and functional characteristics due to a divergence from one of the physiological stages of progressive wound healing [1,2]. In the 1950s, the phrase “chronic wound” was first used in the literature to describe wounds that were challenging to cure or did not proceed according to a typical healing pattern. The phrase has drawn criticism, though, because it is unclear how long chronicity lasts. Alternative terms including difficult-to-heal, non-healing, and complex wounds have been proposed.

Chronic wounds are commonly defined as “wounds that have not undergone orderly and timely repair to produce anatomic and functional integrity after 3 months”. Recent reviews have also emphasized the need for additional research in this area and the lack of agreement on the definition of “chronic wound” [2]. Most wounds experience unhindered hemostasis, inflammation, proliferation, and maturation during the normal healing process but a portion will miss these steps, leading to chronic wounds with associated morbidity and expense. The management of a wound to encourage natural healing or to facilitate alternative methods of healing is known as wound bed preparation. Promoting the chance of healing is especially important when evaluating chronic wounds systematically. The accumulation of necrotic tissue that occurs as a result of chronic wounds must be treated to promote healing. To speed up the healing process, necrotic tissue is removed from the wound bed, pressure is released, the underlying tissue is examined, dead spaces that could harbor bacteria are removed, pus is drained, and topical preparations are optimized [3].

Chronic wounds are frequently made more difficult by a variety of barriers that appear during the healing process, such as ischemia, necrotic tissues, bacterial charges, or an abundance of matrix metalloproteinases that promote inflammation. Such factors prolong the inflammatory phase of wound healing while continuing to recruit macrophages and neutrophils inside the wound. The high levels of inflammatory metalloproteinases recorded by such types of wounds are associated with the continuous destruction of the extracellular matrix. Apart from these high levels of inflammatory proteases, the low levels of growth factors are also characteristic of chronic wounds [1,3].

Atonic wounds are frequent, as they are usually incorrectly treated. Morbidity and the costs associated with the therapy of such wounds call for the implementation of some prevention and treatment guides [4,5,6]. The common wounds of the low extremities include arterial, diabetic, pressure, and venous ulcers, so it is only the physical examination that usually can guide the diagnostic [5]. All patients with unhealed lower extremity ulcers should undergo vascular evaluation, including documentation of wound location, size, depth, drainage, and tissue type [1,6]. A major problem of global health is that of chronic wounds, for which an adequate diagnosis and treatment are essential, equally for their healing and for preventing possible subsequent complications [1]. Each wound should be treated individually as to its cause, chronicity, location, and level of microbial contamination. Traditional methods of diagnosis and treatment of chronic wounds have shown limited effectiveness, which requires the development of innovative diagnostic and treatment procedures for their healing. The patients suffering from such types of wounds complain of serious physical, mental, and social consequences that they experienced [3,7,8,9].

Utilization of modern and advanced technologies for more rapid wound healing may substantially reduce the costs. Image analysis of a wound area represents extremely valuable information. The recent discovery of neural networks and deep learning algorithms has led to considerable improvement in performance in various fields, which has led to a significant increase in research work in the field of wound analysis as well [10].

While chronic wounds are characterized by local inflammation that participates in the healing process and may take months to heal, atonic wounds lack inflammatory markers such as neutrophil infiltration and generally do not heal [11].

Evaluation and study of some new therapies are of major importance for facilitating the surgical intervention, as well as for a more rapid recovery of the patient by means of less invasive or even non-invasive procedures [11,12,13]. The prevention of chronic infection of a wound involves numerous strategies and methods, which are applied either simultaneously or alternately between them.

Accordingly, depending on the indications received, various methods are applied, such as mechanical washing and cleaning, application of antiseptics, debridement, vacuum-assisted closure of the plague, moist healing by oxygenation through active and passive compresses, removal or destruction of germs present at the level of the wound [12,13,14,15,16,17].

The laser therapy of chronic wounds includes the application of an infrared light upon the damaged areas, for stimulating and improving the healing of soft tissues, which leads to the melioration of both acute and chronic conditions [17,18,19,20]. The laser therapy stimulates microcirculation at the local level, while improving the lymphatic drainage of the pathological zone. Combining biostimulation and photomechanical stimulation, laser therapy heals the tissue, concomitantly offering an intense and non-dependent form of pain management [21,22,23,24]. A high-intensity laser produces photochemical, photothermal, and photomechanical actions with a sufficient dose to reach the deep target tissue, which has a positive effect on reducing pain by penetrating deep tissues and providing functional improvement [25].

High-intensity laser therapy is better than low-level laser therapy which takes much longer to deliver the same amount of energy to the tissues.

High-intensity lasers, which are more potent, can penetrate more deeply than low-level lasers. Additionally, high-intensity lasers can treat a larger area than low-level lasers. This enables the healing light’s energy to reach the supporting tissues around the injury as well as the injured area [25,26,27].

The aim of the present investigation is to provide information on the healing process through the application of laser therapy at the plague level.

## 2. Materials and Methods

### 2.1. Aim of the Study

The purpose of the study is to highlight the beneficial effect of using the laser in atonic wounds. The study is an observational study, a longitudinal case study, on the first and the 21st day after treatment.

### 2.2. Materials

The study has been developed, between 1 July 2020–31 July 2021, in the Surgery Clinics of the CF Clinical Hospital of Oradea, on a total number of 95 patients suffering from various pathologies, with which the presence of atonic wounds had been associated. We collected data from the reference group and study group upon hospitalization (day 0), day 3, day 7, and the 21st day after treatment.

The subjects were divided into 2 groups: an experimental one—a study group, including 65 patients whose wounds were treated by laser therapy; and a reference one—a reference group, formed of 30 patients to whom no laser had been applied, and their treatment involved both medicines—antibiotics, anti-inflammatory, and analgesic drugs—and a local cure, namely, a strict daily dressing with various antiseptic compounds. Both male and female subjects, with ages between 30 and 70 years, were included. A type laser with a 12-watt laser probe was used in a 1064 nm emitting program. The high power of up to 12 W allows deep tissue penetration in continuous mode and pulsatile mode for immediate pain relief, stimulates local microcirculation, and supports lymphatic drainage of the pathological area by precisely defining the area to be treated. Combining biostimulation and photomechanical stimulation, high-intensity laser therapy actually heals tissue while providing a powerful, non-addictive form of pain management. The therapeutic effect is major by using a wavelength of 1064 nm.

Most cases were reported in the age group between 51–60 years, followed by the age group between 41–50 years.

Following the comorbidities of the patients in the study, we claim that no patients with serious diseases or immunosuppressed patients were included, but patients with comorbidities that influence the general evolution less, such as heart valvular insufficiency in different stages, heart valve stenoses in different stages, arterial hypertension essential of various degrees, chronic ischemic cardiopathy, hydrostatic varicose veins, and congestive heart failure. All these comorbidities are with chronic treatment and are being monitored. Regarding the history of the wounds, the patients who have been hospitalized have a history of general drug treatment with antibiotics, some with tinnitus antibiotic therapy, and wound antiseptics with various substances such as the application of various local topicals, but with an evolution.

The patients gave their written consent for inclusion in the study and the agreement was signed by each patient at the time of admission, each of the patients taking note of the general aspects and treatment as well as the purpose of the study.

The main inclusion criterion was the presence of a chronic pathology such as chronic arteriopathy obliterans and diabetes mellitus as well as peripheral vascular disease, pathologies in which the wounds have a long evolution and negatively influence the daily routine of the patients and who signed the agreement to participate in the study.

The exclusion criteria refer to patients of different ages, to the presence of chronic wounds of a traumatic/contusion nature, as well as to other conditions preventing participation, such as limited cooperation, severe diseases which affect the living conditions (cancer, advanced renal insufficiency, and advanced hepatic insufficiency), or patients have not given their consent.

The present study observed the ethical conditions established by the Helsinki Declaration, being approved by the local ethic committee of CF Clinical Hospital of Oradea, nr. 2627/15.06.2020.

### 2.3. Methods

Laser therapy was applied to the wound through an applicator with a 30 mm diameter protective spacer. The intensity was distributed evenly over 8 min and 20 s at the level of the wounds. Upon contact with the skin, the laser light creates a wavelength-specific photomechanical wave in human tissue.

Our study also involved a reference batch, formed of 30 patients, to whom no laser therapy had been applied for chronic wounds. A questionnaire was addressed to the patients which included a series of questions related to the intensity of pain, discomfort when walking, discomfort felt during the night period, a moment of injury appearance, and previous treatments for the current pathology.

Their treatment involved both medicines—antibiotics, anti-inflammatory, and analgesic drugs—and a local cure, namely, a strict daily dressing with various antiseptic compounds, but no laser therapy. This permitted an observational, comparative study between the groups with no laser utilization versus that in which laser therapy had been applied.

Tissue samples were taken and fixed in 10% buffered formalin (pH 7.4) for up to 72 h and embedded in paraffin according to standard procedures. After that, the tissue was stained by H&E the cases were analyzed by two qualified pathologists.

### 2.4. Statistical Analysis

The data were statistically analyzed using SPSS 26. Statistical significance was considered at the standard 0.01. Differences between the study group and the control group were analyzed using a *t*-test, *p* < 0.01.

## 3. Results

Such pathology was manifested in some patients earlier, even if they went to the doctor for another medical problem, but its presence was ignored. A careful anamnesis showed that, in almost half of such patients, the wound had been present for at least 3 months, but it had been ignored and treated with various local topics, with no beneficial effect upon healing. Correlated with the delayed visit to the physician and with the combined application of various topical substances in the attempt of healing the wound without any medical assistance, mention should be also made of the involvement of certain hygienic–dietetic–social factors with negative influence on healing.

**Figure 1:**
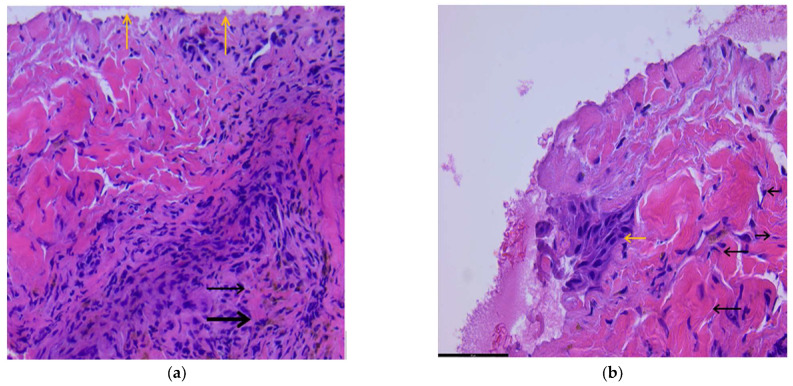
(**a**) During laser treatment; (**b**) after laser treatment, the presence of a small area of re-epithelialization with squamous cells (yellow arrow), located superficially, and numerous fibroblasts (cells with a cigar-shaped nucleus—black arrow).

With laser treatment, the ulceration of the skin with areas of necrosis (superficial) is observed, and aspects of regeneration are noted. Connective tissue fibers, vessels, and numerous siderophages are present, as shown in Figure 1a, 100 HX.

After the laser treatment, the presence of a small area of re-epithelialization with squamous cells (yellow arrow), located superficially, and numerous fibroblasts (cells with a cigar-shaped nucleus—black arrow) are present underneath, which denotes an active regeneration phenomenon. Numerous collagen fibers are still visible, which will ensure the healing process, as shown in Figure 1b.

In the study group, the laser treatment of atonic wounds was proven to be efficient with a positive impact.

### 3.1. Characteristics of the Population

In our study group over 61.5% were women, the age was between 30–70 years, the average age was 60.18 years, and the patients came mainly from the rural environment (57.47%), the urban/rural ratio being1: 1.4, Table 1. Also, the differences are considered to be not statistically significant between the demographic characteristics of the study and reference groups, (*p* > 0.001).

**Figure 2:**
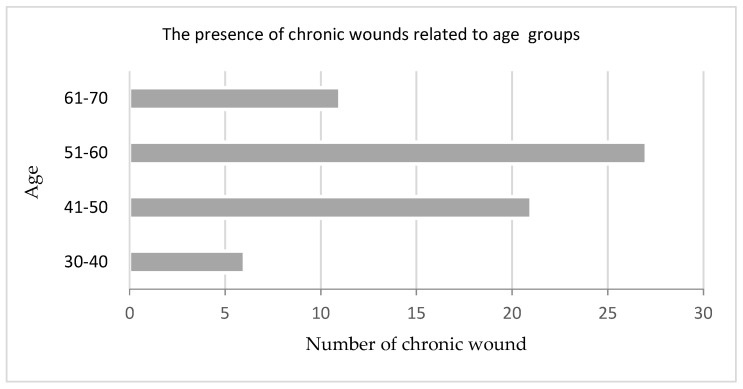
Patients by age and number of chronic wounds.

Interpretation of the questionnaire filled in by the patients shows that, in the laser therapy batch, 38 patients come from rural areas, while 27 were from the urban milieu. Also observed was that the presence of wounds is more frequent in women, in whom a 61.5% ratio was recorded, comparatively with the 38.5% ratio registered among men, as shown in Figure 2.

As to the reasons explaining the visit to a physician, 31 patients came for a different pathology, the presence of the wound being observed only accidentally, 18 of them came to the surgery department accusing some ulceration, 66.15% are active patients, most of them from rural areas, with a lifestyle requiring no sedentariness.

The examinations performed showed that 33 patients suffered from stage 4b CEAP, chronic obliterating arteriopathy, 18 of them with complicated type II Diabetes mellitus, and 14 subjects—with peripheral venous disease. As to the dominant symptom, namely, the pain felt at the wound level, it was present at the moment of hospitalization in patients of both batches. On hospitalization, 40 of them gave it a score of 8–10 points on a scale from 1 to 10, where 10 expressed pain with impact upon mobilization. The major impact of the laser therapy observed on the intensity of pain shows that 52 of the patients from the study group gave a score of 2–4 points on day 7 post laser therapy, 9 patients gave a score between 4 and 6 points, while 3 patients recorded the same values as during hospitalization.

**Figure 3:**
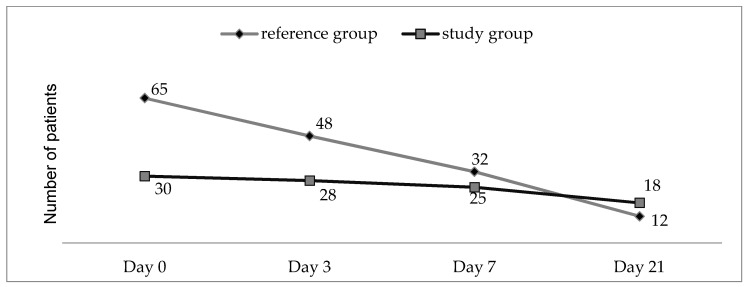
The number of patients who improved their general conditions in study and control group.

A total of 24 patients from the reference group (no laser, only local treatment) gave 5–8 points on the scale of pain, 21 days post-therapy, which evidences the positive impact of laser upon the dominant symptom, namely, local pain, which recorded a considerable reduction after the 21 days of stimulating local treatment. The considerable reduction of the influence of pain on the general condition is much more evident in the treatment of laser wounds after 21 days of treatment, as shown in Figure 3. The number of patients in the study group who improved their general conditions is higher than the control group.

More than 35% of the patients suffered from chronic venous insufficiency, with no antecedents having been manifested, so it was diagnosed only during hospitalization.

Further, 80% of the patients from the study group came to a reference after 4 weeks, an obvious melioration, both in relation to the surface of the wound (30% being in the healing phase) and with the development of their daily activity being registered. Only 10% of them showed aggravation or a stationary evolution of healing; 10% of them did not come to the reference 4 weeks after the laser therapy; 95% of the patients from the reference group showed, 4 weeks later, either an aggravated or a stationary healing process, while 5% of them had an improved local and general symptomatology. Wounds had a much better evolution at younger ages, probably due to tolerance and more prompt response of the body to the wound healing process and stimulation with laser therapy.

Laser therapy is a non-invasive method, so the psychological impact on the patient in the case of wound recovery is a positive one. In the absence of the application of laser treatment, the patient would have additional stress due to the extension of the treatment and hospitalization period. Evaluating the subjective scale, we consider another strong point to be the much faster remission of symptoms under laser treatment.

A disadvantage of this procedure is bleeding at the level of the wound during the laser treatment. Local bleeding was noted during the laser treatment, but for a short time, after which it stopped.

### 3.2. Impact of Laser Therapy on Atonic Wounds

The high-intensity laser therapy of atonic wounds in the study group, compared with the reference one where no such therapy had been used, showed its efficiency and promising positive impact on both symptomatology and local tissue defect. Due to its precision, the impact of laser is of importance, mainly concerning local symptomatology.

A total of 75% of patients in the study group returned to control 90 days after discharge. Of these, 70% did not need another hospitalization for chronic ulcers, 5% required rehospitalization for the same problem, requiring a repeat procedure, and 25% did not come to control for various other reasons.

Regarding the appearance of the wounds, some of the patients presented a minimal local suppuration at admission, from which samples were collected for culture and antibiogram for a targeted anti-biotherapy, the wound being superinfected. The different appearances of the wounds in terms of their size, the general appearance, and the various stages of evolution in which the wound is found at the time of hospitalization differ from case to case. The differences consist of the anatomical location of the wound, the care condition of the wounds at home or in another medical center, the environment of origin, as well as the daily activity of the patients.

**Figure 4:**
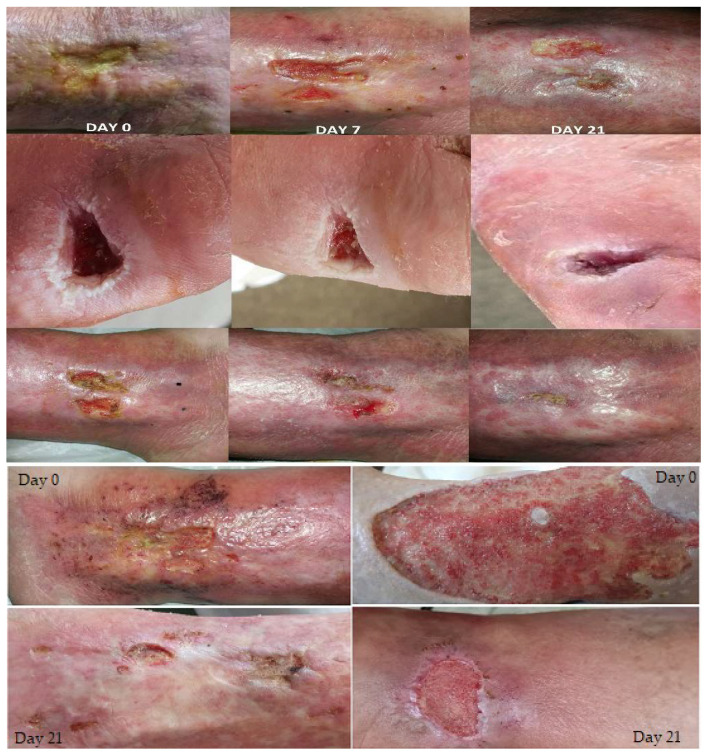
Atonic wounds after laser therapy.

Based on the collected data and an anamnesis and objective examination, we concluded that high-intensity laser therapy benefits the patients under study, stimulating and accelerating the healing process, as shown in Figure 4.

### 3.3. Statistical Analyses

Table 2 shows descriptive statistics that were made between the study group and the reference group showing significant differences between measurements at different times. A significant decrease in pain intensity is observed (*p* < 0.001).

Table 3 shows significant differences between measurements at different times and a significant decrease in discomfort when walking starting from day 3 (*p* < 0.001), as shown in Table 3.

Table 4 also shows a significant decrease in nighttime discomfort starting from day 3 (*p* < 0.001).

**Figure 5:**
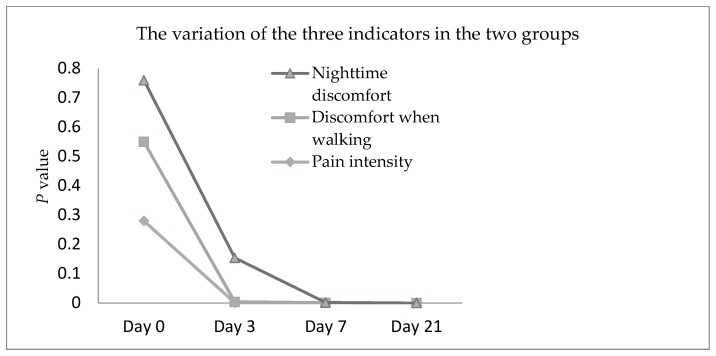
The variation of the three indicators in the two groups.

The statistical analysis of the three indicators taken into account in the questionnaire indicates that, indeed, the study group to which the laser treatment was applied has significant improvements in atonic plaques starting from the 3 days of treatment, as shown in Figure 5.

## 4. Discussion

In the context in which the present study has been performed, namely, the COVID-19 pandemic, the main observation was that patients suffering from chronic wounds avoided consulting a physician for fear of being infected with the SARS-CoV-2, a situation which had a negative impact on their health condition [22,23,24]. The main reason that explained why the subjects avoided a medical service was the fear of being infected with this virus, preferring instead various local, topical, or antiseptic therapies, which, in our opinion, slowed down healing by delaying at the wound level. The main reason why patients affected by this pathology went to a medical center was local pain, especially local nyctalgia [28].

The risk of wound infection increases as local conditions favor bacterial growth. Consequently, the main goal of wound management is to restore the host environment bacterial balance, the most effective method in this sense being the removal of devitalized tissue and foreign bodies from the wound, the reference of bacterial load and inflammation, as well as tissue perfusion [29,30]. Some studies state that laser therapy more effectively stimulates the micro-activation of circulation and tissue metabolism, and has analgesic and vasodilating effects. Such an analgesic effect was also observed in our study, based on the results obtained after the first 7–21 days of local laser therapy [30]. Our patients stated that after 7 days of laser therapy, they noticed positive progress in their general condition, a significant improvement in nighttime sleep, and active mobilization quite similar to normal, with the absence of ulceration. The ulcers of a diabetic leg, one of the most frequent complications of diabetes mellitus, are defined as chronic cutaneous lesions never to be healed or with a prolonged healing time [31,32,33].

Multidisciplinary care of a diabetic leg is quite frequent; however, the results of the treatment are hardly satisfactory. Further studies are necessary for determining the real value of lasers in the routine care of wounds. A comparative analysis of the surface of ulcers caused by diabetic arteriopathy in the two groups under study evidenced a more rapid epithelialization in the patients treated by local laser therapy [34,35].

Our study also shows a beneficial effect, namely, both pain reduction and tissue regeneration in 80% of the subjects, in both the short-term (the hospitalization period) and after that, as evidenced by the results of the reference visits; 10% of them stated that the wound was definitively healed within 90 post-therapy days.

During laser treatment application, in 56.9% of the patients minimum local bleeding occurred on days 5–6 as a result of local vasodilation. Local pain during laser therapy was registered in 89.2% of the subjects on treatment days 3–4, after which it was not felt anymore. In our opinion, such secondary effects of laser therapy are only partial and of short duration, which suggests the pro-healing action of the local therapy. Even if experienced by the patient as a local, partial negative effect, the long-time consequences are beneficial, the symptomatology being attenuated or even eliminated, while the tissue regeneration effect produced epithelialization and final closing of the cutaneous–muscular defect.

The benefit of laser therapy consists of the fact that it is a non-invasive method, so the psychological impact on the patient in restoring the wound is a positive one. The possible risks of laser therapy, such as bleeding or local pain, were manifested only for a short period, both during and after the treatment, but never post hospitalization [36,37,38].

No recrudescence of the infection at the level of the chronic ulceration was observed, as the patients received both antibiotics and local antiseptic drugs. The patients entering the hospital with ulcerations accompanied by suppuration, evidenced by taking a sample of the wound followed by a culture and an antibiogram received targeted antibiotic therapy.

As to the applicability of the treatments at home, considering that some patients tried this before addressing a medical center, the observation made was that the subjects who made use of more than two local topics, or more than two topics associated with various antiseptic substances, had a more severe evolution than those who used less than two topics or an alternating topic/antiseptic treatment, the evolution and result of the laser therapy is better [36,37,38,39].

The topic of the present study was approached because the presence of associated pathologies which cause and maintain chronic ulcerations are more and more numerous, a situation corroborated by a late presentation of the patients to a medical center. The effect on the healing phases is a negative one, as it further delays the healing process along all its stages. In the actual context, a manifestation of the COVID-19 pandemic represented another obstacle in this respect [40,41]. Another limitation of the study is the fact that a biopsy was not performed in the control group at 7 days. The obtained results were not presented because the microscopic changes were similar to those at admission and the macroscopic ones at the level of the wound were minimal with a slow evolution.

Next, 5% of the patients in the experimental group had to be re-hospitalized as their medical problem had not been solved it had become even more serious. That is why a further study of ours will analyze other medical methods which, associated with various—severe or prolonged—comorbidities and combined with laser therapy, might lead to accelerated healing.

The pathology here under analysis was more frequent in female patients and most of the cases occurred in the age group of 51–60 years, followed by 41–50 years. Of all patients, 66.5% of the patients are active persons.

## 5. Conclusions

The effectiveness of the method can be seen in our study with the improvement of symptoms during treatment; the main symptom, local pain, was present in the patients of both batches and the impact of laser therapy was a positive one, accelerating healing if considering that 80% of the subjects showed a significant reduction of the surface of the defect, comparatively with the reference group. Symptomatology was either diminished or even absent 4 weeks after the therapy in the patients of the experimental group and resumption of daily activities was possible after the moment of discharge from the hospital in the study group. Comparatively, with the reference one, 95% of the latter ones asserted either a slight melioration or its absence, which required a re-evaluation of the treatment plan.

## Figures and Tables

**Table 1:** Patient demographics and disease characteristics at baseline visit.

	Study Group	Reference Group	Total	
	n = 65 (100%)	n = 30 (100%)	n = 95 (100%)	*p* Value
Demographic characteristics				
Age in years, mean (SD)	71.24 (15.04)	68.12 (46.15)	69.68 (16.46)	0.9352
Men, number (percentage)	14 (53.84)	12 (46.15)	26 (38.5)	0.9814
Women, number (percentage)	28 (71.79)	11 (28.20)	39 (61.5)	0.8780
Wound characteristics				
Infection	13 (20)	12 (40)	12.5 (13.15)	0.8526
No infection	52 (80)	18 (60)	35 (36.84)	0.0025
Wound area in cm^2^ median (min; max)	3.23	3.44	3.33	0.0020
	(0.24; 24.26)	(0.46; 45.15)	(0.24; 45.15)	
Wound etiology, n (%)				
Peripheral Venous Disease	13 (21.53)	5 (16.66)	3.5 (3.68)	0.0001
Type II Diabetes	18 (27.69)	2 (6.66)	10 (10.52)	0.0001
Chronic Obliterating Arteriopathy	33 (50.76)	23 (76.66)	28 (29.47)	0.3579

**Table 2:** Descriptive statistics for pain intensity in reference and study groups.

Pain Intensity		N	Mean	Std. Deviation	Std. Error Mean	t	df	Sig.
(0—No Pain; 10—Affects)								
Day 0	Study group	65	7.6154	2.15561	0.26737	28.483	64	0.028
	Reference group	30	8.4667	1.35782	0.24790	34.153	29	
Day 3	Study group	65	5.8462	1.83908	0.22811	25.629	64	0.001
	Reference group	30	7.5333	1.30604	0.23845	31.593	29	
Day 7	Study group	65	4.1538	1.75206	0.21732	19.114	64	0.000
	Reference group	30	6.3000	1.55696	0.28426	22.163	29	
Day 21	Study group	65	3.1077	1.84664	0.22905	13.568	64	0.000
	Reference group	30	5.4667	1.73669	0.31707	17.241	29	

Mean—These are the respective means of the variables. df—The degrees of freedom. t—It is the ratio of the mean of the difference to the standard error of the difference. Sig. = This is the *p*-value associated with the correlation. Correlation is significant at the 0.001 level. Std. Deviation—This is the standard deviation of the variable.

**Table 3:** Discomfort when walking in reference and study groups.

Discomfort when Walking (0—Does Not Influence Walking; 5—Impossibility of Walking)		N	Mean	Std. Deviation	Std. Error Mean	t	df	Sig.
Day 0	Study group	65	3.2769	0.91539	0.16713	19.746	29	0.027
	Reference group	30	3.3000	1.25633	0.15583	21.029	64	
Day 3	Study group	65	2.0615	0.77608	0.14169	17.409	29	0.003
	Reference group	30	2.4667	1.10223	0.13671	15.079	64	
Day 7	Study group	65	0.9231	0.62972	0.11497	13.047	29	0.001
	Reference group	30	1.5000	0.87156	0.10810	8.539	64	
Day 21	Study group	65	0.2308	0.79148	0.14450	8.074	29	0.000
	Reference group	30	1.1667	0.49274	0.06112	3.776	64	

Mean—These are the respective means of the variables. df—The degrees of freedom. t—It is the ratio of the mean of the difference to the standard error of the difference. Sig. = This is the *p*-value associated with the correlation. Correlation is significant at the 0.001 level. Std. Deviation—This is the standard deviation of the variable.

**Table 4:** Nighttime discomfort in reference and study groups.

Nighttime Discomfort		N	Mean	Std. Deviation	Std. Error Mean	t	df	Sig.
Day 0	Reference group	30	3.7333	0.98027	0.17897	20.860	29	0.021
	Study group	65	3.5692	1.52037	0.18858	18.927	64	
Day 3	Reference group	30	3.0667	1.22990	0.22455	13.657	29	0.015
	Study group	65	1.9231	0.97320	0.12071	15.931	64	
Day 7	Reference group	30	2.3000	1.26355	0.23069	9.970	29	0.001
	Study group	65	0.8154	0.46410	0.05756	14.165	64	
Day 21	Reference group	30	1.6000	1.06997	0.19535	8.191	29	0.000
	Study group	65	0.1231	0.33108	0.04107	2.997	64	

Mean—These are the respective means of the variables. df—The degrees of freedom. t—It is the ratio of the mean of the difference to the standard error of the difference. Sig. = This is the *p*-value associated with the correlation. Correlation is significant at the 0.001 level. Std. Deviation—This is the standard deviation of the variable.

## Data Availability

Not applicable.
